# Technology Acceptance, Technological Self-Efficacy, and Attitude Toward Technology-Based Self-Directed Learning: Learning Motivation as a Mediator

**DOI:** 10.3389/fpsyg.2020.564294

**Published:** 2020-10-27

**Authors:** Xiaoquan Pan

**Affiliations:** Xingzhi College, Zhejiang Normal University, Jinhua, China

**Keywords:** technology acceptance, technological self-efficacy, attitude, self-directed learning, learning motivation, English language learning

## Abstract

This study explored the contribution of technology acceptance and technological self-efficacy to attitude toward technology-based self-directed learning in a sample of Chinese undergraduate students. The study also inquired into whether learning motivation mediated these associations. A total of 332 undergraduate students of college English course were enrolled to participate in questionnaires regarding their technology acceptance, technological self-efficacy, attitude toward technology-based self-directed learning, and learning motivation. Results indicated that students’ technology acceptance and technological self-efficacy were related to their attitude toward technology-based self-directed learning. The findings also indicated that learning motivation mediated the relations of technology acceptance, technological self-efficacy, and attitude toward technology-based self-directed learning. Specifically, students experiencing greater technology acceptance and technological self-efficacy showed higher attitude toward technology-based self-directed learning. This study highlighted the significance of learning motivation as a mediating mechanism illustrating relations between students’ perception of technology environments and their attitude toward technology-based self-directed learning.

## Introduction

In the wake of network technology, online learning, e-learning, and other informal learning approaches expand resources, venues, and learning spaces, enabling self-initiated construction of learning experience (Lai and Gu, 2011; Reinders and White, 2011). By accessing to the ecology of language learning constructed by technological facilitating conditions, language learners can launch their learning on the basis of their own interests and needs. Thereby, it is indispensable that language learners are equipped with the competence to engage in technology-based self-directed learning (Benson and Reinders, 2011; Reinders and Darasawang, 2012). Researchers have found that undergraduate students do adopt technology for learning (Inozu et al., 2010), but their use of technologies often lacks sufficient effectiveness (Kennedy and Miceli, 2010). Some studies have inquired into a few factors that affect students’ utilization of technology for learning, including competency in technology use (Kennedy et al., 2008), perceived usefulness of technology (Goodyear and Ellis, 2008; Teo, 2011), perception of the utility of technological resources (Clark et al., 2009), and the scaffolding available in supporting the technology-enhanced learning experience (McLoughlin and Lee, 2010). According to Teo et al. (2015, p.78), “attitude toward technology use has been examined in various models that attempt to explain individuals’ intention for technology use, including Technology Acceptance Model (TAM; Davis et al., 1989), TAM2 (Venkatesh and Davis, 2000), and Unified Theory of Acceptance and Use of Technology (UTAUT; Venkatesh et al., 2003).” These studies underscore the importance of understanding of how personal attitude contributes to behavioral intention on technology use. However, there are comparatively few studies of the current literature that elaborate attitudes toward technology use. As such, Tate et al. (2015) posed the concern that current theories are deficient in constructs that could better annotate students’ behavioral intention on technology use from the perspective of attitude. In this study, the author considered enhancing our understanding of the influences that students’ personal characteristics would have on their behavioral intention on technology use. Additionally, confining the understanding only to technology-related factors may thwart a deeper understanding of what influences students’ adopting technology for self-directed learning. Thus, examining this issue by supplementing the variable of learning motivation, this study is expected to add a new perspective to the existing research. Specifically, this study intended to inquire into how technology acceptance and technological self-efficacy contribute to attitude toward technology-based self-directed learning and meanwhile to investigate whether learning motivation mediated these associations in a sample of Chinese undergraduate students studying for college English course.

## Literature Review

### Technology Acceptance and Technological Self-Efficacy

The TAM was first proposed by Davis (1989) on the basis of the theory of reasoned action (TRA) advanced by Fishbein and Ajzen (1975), which is used to explain the associations between the students’ technology acceptance of computer system, behavioral intention, and definite behavior of technology use. Davis (1989) believed that perceived usefulness and ease of use, as antecedent variables, constitute fundamental determinants of users’ technology acceptance and thus affect their actual usage behavior (Teo and van Schaik, 2012). Venkatesh et al. (2003) expanded the TAM with empirical research by adding such factors as social influence, cognitive structure, and experience and the factor of subjective norm that had not been adopted in the original TAM. Using this theory and analysis method, later in the educational landscape, there were studies that verified and explained the intention of students’ technology use (Teo, 2011) and the research of students’ independent use of technology for language learning (Lai, 2013), etc. These studies are the concrete applications of TAM in empirical research. As such, in the educational field, technology acceptance is considered as a prerequisite for learners to adopt information technology to promote learning (Hsieh et al., 2017). Previous studies have discussed learners’ acceptance of different types of technology, such as mobile technology (Pindeh et al., 2016; Nikou and Economides, 2017), computer-based communication technology (Park et al., 2014), social media (Hsieh et al., 2017), and MOOCS courses (Joo et al., 2018). In the technology-supported language learning environment, learners’ perception and acceptance of emerging technologies are important factors that affect their effective learning (McLoughlin and Lee, 2010; Huang and Liaw, 2018) and also one of the core factors that affect self-directed learning (Liaw et al., 2007). Additionally, research on learners’ technology acceptance needs to consider the impact of specific disciplines and social and cultural backgrounds on technology acceptance (Scherer et al., 2019). Related studies also explored the relationship between learners’ technology acceptance and other variables, such as the relationship between technology acceptance and research constructs such as self-regulated learning, self-efficacy, and learning anxiety (Cho and Kim, 2013; Lai, 2013). Liaw and Huang (2013) conducted a relatively successful theoretical innovation by integrating TAM and self-regulated learning theory, revealing the structural relationship between learners’ perceived usefulness, satisfaction, and self-regulated learning.

For many years, Fishbein and Ajzen’s TRA has been conceived as an intentional behavior model for the study of individual behavior associated with information technology. However, Ajzen expanded the explanatory power of TRA in 1991, by adding a new construct of perceived behavioral control, which developed into the theory of planned behavior. In the context of technology-based behavior, several meta-analyses have found a good correlation between individual’s perceived behavioral control and the usefulness of specific technology. Lai (2013) conceptualized perceived behavioral control as “people’s perceptions of their ability and the availability of the support necessary to achieve an expected behavior” (p. 103). Among the widely used, multidimentional constructs of perceived behavioral control, technological self-efficacy was considered as the dominant determinant of the intention of using the technology (Teo, 2009; Teo and van Schaik, 2012). In this study, technological self-efficacy is characterized as students’ perception of their capabilities to utilize technology-related tools and sites to conduct learning behaviors so as to achieve intended learning outcome (Bandura, 1997; Keengwe, 2007). Researchers have verified a significant positive influence of technological self-efficacy on technology acceptance and utilization (Celik and Yesilyurt, 2013) and regarded technological self-efficacy as a proxy of individuals’ control beliefs in technology use (Venkatesh and Davis, 1996). Researchers have also found that technological self-efficacy significantly affects students’ behavioral preferences to use technological tools and their perceptions of the usefulness of technology for learning (Keengwe, 2007; Mew and Honey, 2010). Additionally, Ajzen (2002) decomposed the constructs of perceived behavioral control into two components: controllability and learning motivation. The concept of controllability essentially resembles technological self-efficacy, with both used as a proxy of individuals’ control beliefs in technology use (Venkatesh and Davis,1996), whereas learning motivation means the individual’s judgment of the ability to attain designated types of performance (Bandura, 1986). In the process of technology-based self-directed learning, students’ technological learning motivation is reflected in their mastery and familiarity of technical skills, as is consistent with the study from Mew and Honey (2010), which indicated that technological learning motivation significantly influences students’ intention to use online learning websites, technology-related facilities and their personal technology application. This study conceptualized the technology acceptance and technological learning motivation as supportive and fair. Technology acceptance reflects students’ perceptions that technology is useful and easy to use, and thus they are interested in using it for self-directed learning (Lai, 2013).

### Attitude Toward Technology-Based Self-Directed Learning

The concept of self-directed learning was defined by Knowles (1975) as “a process in which individuals take the initiative, with or without the help of others, in diagnosing their learning needs, formulating learning goals, identifying human and material resources for learning, choosing and implementing appropriate learning strategies, and evaluating learning outcomes”(p.18). The research tradition on self-directed learning emphasized learners’ sense of personal autonomy of holding their learning objectives and assuming ownership of learning (Garrison, 1997; Knowles et al., 2015). For instance, Garrison (1997, p. 18) considered self-directed learning as “an approach where learners are motivated to assume personal responsibility and collaborative control of the cognitive (self-monitoring) and contextual (self-management) processes in constructing and confirming meaningful and worthwhile learning outcomes.” Moreover, studies point to the importance of regarding learners as undertaking self-initiated learning activities (Benson and Reinders, 2011; Reinders and White, 2011). Additionally, a lot of studies on self-directed learning incorporated the dimensions of learning process, which highlighted cognitive and motivational constructs (Garrison, 2003), as well as the learning context and its impact on self-directed learning experiences (Song and Hill, 2007). This is particularly relevant when treating self-directed learning as occurring in a multifaceted and multiple contexts. In the pace of network communication technology, the research on self-directed learning in open educational resource repositories *via* the use of information technology and the Internet (such as MOOCs and online courses) has also received great concern (Kim et al., 2019). Nevertheless, some researchers identified that learners’ active use of technology for language learning does not necessarily guarantee satisfactory outcomes (Lai and Gu, 2011) and does not really reflect a sound understanding of their effective use (Oxford, 2009; Kennedy and Miceli, 2010). Therefore, on the one hand, some external factors, such as computer literacy, technological facilitating conditions, have been viewed as a prerequisite for learners’ effective use of technology (Hubbard and Romeo, 2012); on the other hand, learners’ willingness to engage in technology use for self-directed learning has been highlighted ((Kop and Fournier, 2011). Some educational research intended to enhance self-directed learning incorporated multifaceted components that predict learners’ active engagement in technology use. Attitude was argued to be very relevant to students’ voluntary utilization of technology for learning (Saadé and Galloway, 2005). For instance, Lai (2013, p. 115) examined “three major attitudinal factors that drove the participants’ willingness to use technology for language learning: intended learning effort, perceived usefulness of technology for language learning, and perceived educational compatibility of technology with language learning needs and preferences.”

Previous studies (e.g., Teo, 2011; Teo and Wong, 2013) highlighted that students’ beliefs on the utility of technology influenced attitude toward technology use. While attitude toward technology use was regarded as an individuals’ overall affective response to using technology system, representing individual’s emotional experience associated with technology use (Venkatesh et al., 2003). In this study, attitudes toward technology-based self-directed learning represent undergraduates’ overall affective responses to utilizing technology in English language learning. In the TAM (Davis et al., 1989), individuals’ attitude toward using technology (Teo, 2010 2012; Jan and Contreras, 2011) was significantly predicted by perceived usefulness and ease of use of technology, which was in turn hypothesized to affect their behavioral intention to use technology and actual use.

Previous studies support the notion that students’ perception of technology environments constitutes an important element for their academic-related beliefs. Specifically, students perceiving the convenience and availability in their interactive learning process report higher motivation, engagement, and persistence in learning (Wentzel et al., 2010; Tas, 2016). Some empirical evidence also indicated that students’ perception of the technology environments is linked to attitude toward technology-based self-directed learning. However, the previous studies on technology use and its influencing factors mainly build on cross-sectional study. Importantly, in the current literatures, longitudinal studies are insufficient; thus, little is recognized about how earlier technology acceptance and technological self-efficacy in the learning process are associated with students’ attitude toward technology-based self-directed learning later. Thus, the relation between students’ perceptions of technology environments and later attitude toward technology-based self-directed learning deserves further investigation.

To extend the literature, the fundamental aim of our study was to inquire into the relation between students’ acceptance of technology environments, technological self-efficacy, and their attitude toward technology-based self-directed learning. Based on previous literature (Kop and Fournier, 2011; Lai et al., 2012), this study hypothesized that students who perceive greater technology use would report higher levels of attitude.

### Learning Motivation as a Mediator Between the Technology Acceptance and Technological Self-Efficacy and Attitude Toward Technology-Based Self-Directed Learning

Learning motivation is the sum of the incentives that positively force the choice of a specific behavior or purpose (Jarvis, 2005). As a major psychological concept, motivation is widely believed to be an important factor contributing to students’ acquisition outcomes of second or foreign language (Lamb and Arisandy, 2019). One leading psychological theory of motivation that was typically applied in language acquisition and cognition is the self-determination theory (SDT) put forward by Deci and Ryan (2000). SDT concentrates largely on how environments support or thwart people’s basic psychological needs for autonomy, competence, and relatedness (Jeno et al., 2019). “From a SDT perspective, individual motivation is defined as the degree of autonomy that individuals display during learning activity, and it falls into two major motivational orientations: (1) self-determined forms of intrinsic motivation; and (2) controlled forms of extrinsic motivation (Gan, 2020, p. 3).” Therefore, SDT constructed a theoretical foundation for the motivation process about individual self-determination behavior, stipulating that the environment enhances the internal motivation and promotes the internalization of the external motivation by satisfying the basic psychological needs of the individual. Therefrom, the study of learning motivation was shifted from the understanding of the internalization process of learning motivation to creating an environment conducive to self-determination, initiating a new perspective on the follow-up study of learning motivation. According to Deci and Ryan (2000), intrinsic motivation helps to construct students’ experience of pleasure, enjoyment, and satisfaction, which in turn would further motivate their learning engagement (Dysvik and Kuvaas, 2013). In the present study, SDT has shaped our view of learning motivation. Intrinsically motivated students in technology-based self-directed learning not only seek external technology-enhanced resources but also develop idiosyncratic cognitive intention (Stafford et al., 2004). Extrinsic motivation mainly focuses on the desired consequences that learners behave to achieve (Dysvik and Kuvaas, 2013). Significantly, related studies have conformed to the positive and strong associations between intrinsic motivation and extrinsic motivation (Gonzales, 2011). Technology-based self-directed learning, as an activity and event for learners to undertake their own learning responsibilities (Perry and Winne, 2006), not only entails the accessibility of technology but more importantly the acceleration of learning motivation. “This is because students today are becoming more complex, requiring the researcher to look beyond technology-related enablers (e.g., motivation, social; Hashim et al., 2015, p.383).” Mercer (2011) considered self-directed language learning behavior to be contingent on “a learner’s sense of agency involving their belief systems, and the control parameters of motivation, affect, metacognitive/self-regulatory skills, as well as actual abilities and the affordances, actual and perceived in specific settings” (p. 9). As motivation is acknowledged as the internal force and decisive factor to induce, promote, and maintain individual learning activities, a number of researchers have conceptualized the theories of learning motivation and explored the contribution of learning motivation to students’ readiness, willingness, and intention to use technology for learning. Chiu et al. (2007) analyzed the antecedents of web-based learning continuance, finding that students’ technology-based learning intention was mediated through their satisfaction with technology use for learning. Knowles and Kerkman (2007) identified the linkage between learning motivation and attitude when students are engaged in online learning. Based on TAM and motivational and social-cognitive frameworks, Ifinedo (2017) identified that students’ intrinsic motivation and attitudes toward blog use significantly determined students’ intention to continuously utilize blogs for learning. Additionally, Romero-Frías et al. (2020) explored how motivation influences students’ participation in MOOCS and how they are associated with technology acceptance variables. Under the background of diverse learning resources and channels, the external technological conditions could better accommodate students’ emerging learning needs. Technological learning motivation is characterized with learners’ perception of their capabilities to use technology to execute courses of actions to achieve intended outcome (Compeau and Higgins, 1995), is argued to have a significant positive influence on technology acceptance and use (Straub, 2009), and has been used as a proxy of individuals’ control beliefs in technology use (Venkatesh and Davis, 1996).

Technological learning motivation highlights that students’ attitude toward technology-based self-directed learning is related to satisfying their basic psychological needs of competence and regulate their behavior in the achievement-related context. Despite the evidence on the importance of students’ perceptions of technology climate on their attitude toward technology-based self-directed learning, less is known about the mechanisms through which the technology acceptance and technological self-efficacy affect the students’ attitude toward technology-based self-directed learning. Thus, to extend the literature, this study tested whether learning motivation mediated the links of students’ perception of technology acceptance and technological self-efficacy with their attitude toward technology-based self-directed learning.

Few studies explored whether learning motivation may explain the associations between the technology acceptance and technological self-efficacy and students’ attitude toward technology-based self-directed learning, and none of previous studies assessed the simultaneous role of technology acceptance and technological self-efficacy. Grounded on the previous literature, this study anticipates that learning motivation would be positively correlated to technology use and learning attitude. Further, it is expected that the perception of technology climate would predict students’ perception of learning motivation, which in turn would predict their attitude toward technology-based self-directed learning. Few previous studies exploring the relation between technology climate and students’ perceived attitude toward technology-based self-directed learning have been conducted in samples of students from Asian countries, especially China (e.g., Teo, 2009; Lai et al., 2012). To enhance the literature in the Asian countries, including China, which are characterized by different social and political ideology (Schwartz, 2006), a sample of Chinese undergraduate students was recruited to participate in the study, aiming to explore the specific relations between the perception of technology acceptance and technological self-efficacy and students’ learning motivation and attitude toward technology-based self-directed learning in Eastern Asian cultural contexts.

### Research Questions

Informed by the above discussed new visions in technology use for educational research, the overarching research questions for the present study are as follows:

1. What are the contributions of technology acceptance and technological self-efficacy to attitude toward technology-based self-directed learning?
2. Will learning motivation mediate these relationships?

## Methodology

### Participants

A total of 332 freshmen students (118 boys, accounting for 35.5%) studying for a college English course in the university where the author works in Eastern China participated in the study. Noticeably, in China, college English involves the exclusive use of the English as a second language as the medium for instruction and learning and is a compulsory course for undergraduate students for a minimum of 2 years. Nowadays, as network technology advances, college English teaching and learning initiate full utilization of technology, especially for language learning beyond class.

### Procedure

This study comprised three steps. For step 1, at the beginning of the semester in September, 332 freshmen students from six classes of college English course were instructed and introduced into utilizing available technologies to conduct self-directed language learning beyond class. For step 2, at the end of the semester in January, the participants filled in the hard-copy questionnaire regarding technology acceptance, technological self-efficacy, and attitude toward technology-based self-directed learning for an anonymous survey. At intervals before class, the questionnaire was distributed to all the 332 freshmen in the classroom, answered on the spot, and recycled immediately. Students’ participation was cooperative and voluntary, and thus they carefully completed the questionnaire. All the collected 332 questionnaires were valid, with a 100% completed rate. All the research data collected were anonymized to protect participants’ privacy. For step 3, at the end of the second semester in July, the 325 students completed the hard-copy questionnaire regarding learning motivation as they did in the second step; the 7 absent students completed this questionnaire through the second round of supplementary procedures, and thus, in total, 332 valid samples were collected.

### Measures

#### Technology Acceptance and Technological Self-Efficacy

The survey questionnaire that was validated from previous studies in educational settings (e.g., Davis, 1989; Teo, 2009) was used to assess students’ perceptions of technology acceptance and technological self-efficacy. Technology acceptance was measured using two scales: perceived usefulness (seven items, e.g., technology use helps expand learning opportunities) and perceived ease of use (four items, e.g., the use of technology does not require many instructions). Technological self-efficacy was assessed using five items, e.g., I know how to use technology on my own. A six-point Likert scale was used for the questionnaire items, ranging from 1 (strongly disagree) to 6 (strongly agree). Higher scores indicated higher perceptions of technology acceptance and technological self-efficacy. The standardized factor loadings (SFLs) of the 16 items of technology acceptance and technology self-efficacy range from 0.804 to 0.940, and the Cronbach α values of technology acceptance and technological self-efficacy are 0.898 and 0.879, respectively. In addition, the Kaiser-Meyer-Olkin (KMO) value for validity is 0.918 and 0.907, respectively, indicating that the questionnaire has a good reliability and validity. Finally, the confirmatory factor analysis (CFA) was conducted to determine the validity of Technology Acceptance and Technological Self-Efficacy as an entire scale. Satisfactory model fits were found with χ^2^/*df* = 2.459, Tucker-Lewis index (TLI) = 0.952, comparative fit index (CFI) = 0.962, root mean square error of approximation (RMSEA) = 0.067, and standardized root mean residual (SRMR) = 0.049.

#### Attitude Toward Technology-Based Self-Directed Learning

The questionnaire of attitude toward technology-based self-directed learning was adapted from Compeau and Higgins (1995) and Saadé and Galloway (2005). The questionnaire contained eight items. A sample item is “I am keen on using technologies to facilitate self-directed language learning.” Participants rated the degree of conformity with their attitude toward technology-based self-directed learning using a six-point Likert scale, ranging from 1 (strongly disagree) to 6 (strongly agree). The SFLs of the eight items range from 0.828 to 0.848, the Cronbach α values are 0.897, and the KMO value for validity is 0.912, indicating that the questionnaire has a good reliability and validity.

#### Learning Motivation

In this study, the motivation factors described in Guilloteaux and Dörnyei (2008) and Kormos and Csizer (2014) were used, some of which were revised and developed in combination with the actual situation. A six-point Likert scale was used, and the participants were required to select according to the actual degree of compliance, from 1 “very inconsistent” to 6 “very consistent.” Initial CFA revealed that factor loadings of two items (“I was ready to work hard at English through technology use” and “I really enjoyed learning English through technology platforms”) were low. After the two items with weak factor loadings were removed, the CFA of the remaining 16 items got satisfactory model fitting: χ^2^/*df* = 1.793, TLI = 0.953, CFI = 0.952, RMSEA = 0.057, and SRMR = 0.062. The scale items included the following: (1) confidence and effort (seven items), (2) English language learning interest (four items), and (3) motivation to achieve learning goals (five items). The overall Cronbach α is 0.913, indicating a good reliability.

### Method of Data Analysis

In this study, structural equation modeling was used, and a two-stage approach to data analysis was adopted (Anderson and Gerbing, 1988). The first step is to analyze the measurement model, which defines the relationship between the latent structure and the observed measurement factors. The second step is to analyze the structural model, which specifically defines the relationship among latent structures. Amos 21.0 was used to analyze the model, and a variance-covariance matrix as input and maximum likelihood as the method for estimation was adopted.

Several fitting indices were used to evaluate the overall model fit. Because the χ^2^ test was highly sensitive to the sample size, the ratio of χ^2^ to its degree of freedom (χ^2^/*df*) was calculated. For a model to be assessed as a good fit, the χ^2^ normalized by degrees of freedom (χ^2^/*df*) should not exceed 3.00 (Carmines and McIver, 1981). In addition, TLI, CFI, RMSEA, and SRMR were used. Hu and Bentler (1999) suggested that TLI and CFI should be greater than or equal to 0.90 to indicate good suitability, and RMSEA and SRMR should be less than 0.06 and 0.08, respectively.

In addition, the significance of the mediation effects was assessed using the bias-corrected percentile bootstrap method (Hayes, 2013), computing the confidence interval (CI) for the mediated effect. When zero is not in the CI, it indicates the significance of the indirect effect; thus, the effects of the technology acceptance and technological self-efficacy on the attitude toward technology-based self-directed learning are mediated by learning motivation.

## Results

### Demographic Information

In the demographic descriptions in the questionnaire, the mean age of the participants was 18.48 (SD = 0.55) years, and the duration of technology-based self-directed learning was specifically reported into learning when interested (76 students, accounting for 22.9%), less than 2 h per week (105 students, accounting for 31.6%), 3 to 6 h per week (90 students, accounting for 27.1%), and more than 7 h per week (61 students, accounting for 18.4%), and the used technology platforms (multiple choice) were reported to be as follows: mobile phone (280 students, accounting for 84.3%), our school’s network resources (105 students, accounting for 31.6%), MOOC courses in Chinese universities (102 students, accounting for 30.7%), and other website platform resources (203 students, accounting for 61.1%).

### Descriptive Statistics and Correlations

Table 1 presents descriptive statistics of the main study variables. The participants’ gender did not significantly correlate with attitude toward technology-based self-directed learning, *r* = 0.08; *p* > 0.05.

**Table 1:** Descriptive statistics of study variables.

	*n*	Minimum	Maximum	Mean	SD
TA	332	1.60	6.00	5.26	0.74
TSE	332	1.67	6.00	4.63	0.98
LM	332	3.00	6.00	4.85	0.85
ATSL	332	2.60	6.00	5.06	0.76

TA, technology acceptance; TSE, technological self-efficacy; LM, learning motivation; ATSL, attitude toward technology-based self-directed learning.

All the measures had acceptable reliabilities (ranged from 0.879 to 0.913). Pearson correlation matrices for the relations between variables are displayed in Table 2, indicating that there are significant correlations among the study variables. But none of the correlation coefficients exceeded 0.80, excluding the issue of multicolinearity (Tabachnick and Fidell, 2007).

**Table 2:** Correlations among study variables.

S. No	Variables	1	2	3	4
1.	TA	(0.898)			
2.	TSE	0.306^**^	(0.879)		
3.	LM	0.516^**^	0.496^**^	(0.913)	
4.	ATSL	0.593^**^	0.427^**^	0.703^**^	(0.897)

N = 332. TA, technology acceptance; TSE, technological self-efficacy; LM, learning motivation; ATSL, attitude toward technology-based self-directed learning. Reliabilities (Cronbach α) are shown on the diagonal in parentheses.

** *p* < 0.01.

### Test of the Measurement Model

The quality of the measurement model was tested *via* CFA. Convergent and discriminant validities were established by examining *t* value (CR > 2), the significance of individual item loadings, SE value (>0) of parameter estimation, and average variance extracted (AVE > 0.50). According to Teo and van Schaik (2012), convergent validity, which examines whether individual indicators are indeed measuring the constructs they are purported to measure, was assessed using standardized indicator factor loadings, and they should be significant and exceed 0.7, and AVE by each construct should exceed the variance due to measurement error for that construct (i.e., AVE should exceed 0.50). The results of the data analysis in this study indicated that the SFL of all items of the constructs exceeded the minimum value of 0.70, and the AVE values ranged from 0.710 to 0.835, far higher than the threshold value of 0.50. Hence, this measurement model in this study established the convergent validity of all the measurement items. In addition, the test result of discriminant validity, which assesses whether individual indicators can adequately distinguish between different constructs, displayed that the square root of AVE of each construct was much higher (0.846–0.913) than corresponding correlation matrix (0.306–0.703) for that variable in all cases, thereby ensuring discriminant validity (Teo and van Schaik, 2012). Finally, there was adequate model fit for the measurement model, χ^2^/*df* = 2.665, TLI = 0.953, CFI = 0.967, RMSEA = 0.071, and SRMR = 0.047, indicating that the items were reliable indicators of the hypothesized constructs, thus allowing tests of the structural relationships in the various models to proceed (Teo and van Schaik, 2012).

### Path Analysis Testing the Hypothesized Model

This study adopted Amos 21.0 to test the hypothesized model of Chinese undergraduate students’ attitude toward technology-based self-directed learning in order to verify the influence of various factors and modify the hypothesis model according to preliminary test results. Compared with the modified hypothesis model, the unrevised hypothesis model contained the path of technological self-efficacy → attitude toward technology-based self-directed learning. The verification results showed that the standardized path coefficient is 0.065, SE = 0.032, CR = 1.812 (<2), *p* = 0.155 (>0.05), indicating that technological self-efficacy has no significant impact on attitude toward technology-based self-directed learning, so this study deleted this path and tested the modified model again.

**Figure 1:**
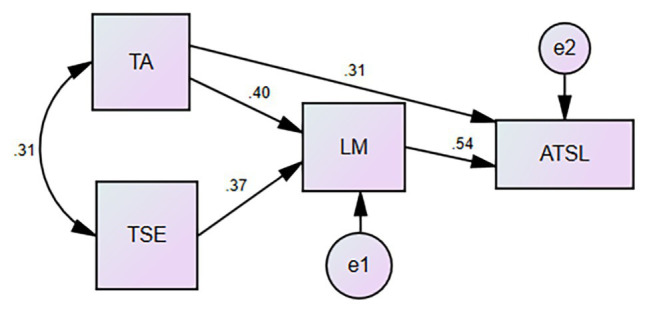
Path analysis of the hypothesized model (*n* = 332). Standardized path coefficients are reported.

The modified structural equation model (Figure 1) has a better fit. Table 3 demonstrated that the standardized path coefficient is not close to or greater than 1, and the parameter estimation SE value is greater than 0, indicating that the parameters of the structural model are reasonable; the CR critical value is greater than 2, and the *p* value is significant at the level of 0.001, indicating that the parameters of the structural model are significant.

**Table 3:** Testing results of the modified hypothesis model.

Path	Path coefficient	SE	CR	*p*
TA → LM	0.402	0.052	8.895	***
TSE → LM	0.373	0.039	8.256	^***^
LM → ATSL	0.542	0.038	12.784	^***^
TA → ATSL	0.313	0.044	7.391	^***^

Path coefficient = standardized path coefficient.

*** *p* < 0.001.

TA, technology acceptance; TSE, technological self-efficacy; LM, learning motivation; ATSL, attitude toward technology-based self-directed learning.

As shown in Table 4, the CMIN/DF value of the modified model is 2.986 (<3), indicating that the fitting value is better, and all the parameters (SRMR = 0.038 < 0.05, RMSEA = 0.075 < 0.08, CFI = 0.994 > 0.90, TLI = 0.962 > 0.90) meet the requirements of the fitting standard value. Therefore, this study considered that the modified model has a good fit.

**Table 4:** Comparison of fitting test value and fitting standard value of the modified hypothesis model.

	CMIN/DF	SRMR	RMSEA	CFI	TLI
Fitting standard value	<3 is better, <5 is acceptable	<0.06	<0.08	>0.90	>0.90
Fitting test value of the modified hypothesis model	2.986	0.038	0.075	0.994	0.962

CMIN/DF = Chi-square/Degrees of freedom.

Technology acceptance significantly predicted attitude toward technology-based self-directed learning (β = 0.313, *p* < 0.001) and learning motivation (β = 0.402, *p* < 0.001); technological self-efficacy significantly predicted learning motivation (β = 0.373, *p* < 0.001), and learning motivation significantly predicted attitude toward technology-based self-directed learning (β = 0.542, *p* < 0.001).

### Assessment of Mediating Paths

The results indicated that learning motivation mediated the relation of technology acceptance with attitude toward technology-based self-directed learning: estimate = 0.462, SE = 0.052, 95% CI (0.177–0.292), indirect effect = 0.224; and the relation of technological self-efficacy with attitude toward technology-based self-directed learning: estimate = 0.323; SE = 0.039; 95% CI (0.106–0.212), indirect effect = 0.157, respectively.

## Discussion

In this study, there were more female respondents (64.5%). The analysis of variance conducted to examine the mean differences between male and female in their decision to utilize technology for self-directed learning showed no significant gender differences, which is consistent with the previous study from Perse and Courtright (1993). This indicated that gender does not influence students’ decision to adopt technology for self-directed learning as they are equally motivated.

The demographic descriptions in the questionnaire demonstrated that Chinese undergraduate students had experience in adopting technology for self-directed learning purposes and had preferences to diverse technology platforms. From the findings, undergraduate students are more likely to adopt the technological medium that is able to tally with their technology acceptance and technological self-efficacy (e.g., mobile phone). This accords with the previous study from Mondi et al. (2008), who suggested that technology used to support learning should not be too complicated and able to allow them to have positive personal fulfillment toward knowledge construction during the learning process. Besides, the less utilization of both school’s network resources (31.6%) and MOOC courses in Chinese universities (30.7%) demonstrated by this questionnaire survey highlighted the issue of educational compatibility, as previous studies have established compatibility as an important predictor of information system acceptance (Hardgrave et al., 2003; Liao and Lu, 2008).

This study explored the contribution of two individual characteristics—technology acceptance and technological self-efficacy—to attitude toward technology-based self-directed learning. This study also expanded previous research by assessing whether students’ learning motivation mediated the relation between students’ perceptions of technology use and their attitude toward technology-based self-directed learning. Specifically, it tested whether the technology acceptance and technological self-efficacy predicted students’ perception of learning motivation, which in turn is associated with attitude toward technology-based self-directed learning.

Correlational analyses corroborated the links between technology acceptance and technological self-efficacy and attitude toward technology-based self-directed learning, which is consistent with previous studies (Teo, 2011; Lai, 2013). Specifically, students perceiving usefulness and easy use of technology in after-class self-directed learning also report higher attitude toward technology use. Additionally, the results of the path analysis by assessing the simultaneous influence of technology acceptance and technological self-efficacy together with the effects of other variables (e.g., learning motivation) involved demonstrated that technology acceptance and technological self-efficacy have a unique contribution to students’ attitude toward technology-based self-directed learning. These results complement those previous studies that typically assessed related variables on the basis of TAM (e.g., Teo, 2009). Further, this study added to the current literature by adopting Chinese adolescent sample, indicating that, for Chinese undergraduate students, the perceptions of technology acceptance and technological self-efficacy impact on later attitude toward technology-based self-directed learning.

Although previous studies highlighted the links of related variables of students’ technology acceptance (Teo, 2009; Lai, 2013), the other generating constructs (e.g., learning motivation) that may affect these associations have not been revealed. This study attempted to explore the latent effect that learning motivation may exert on explaining these associations. Initially, it examined how technology acceptance and technological self-efficacy were related to students’ learning motivation. Results indicated that students who perceived greater technology acceptance and self-efficacy reported increased perception of learning motivation. These findings confirmed that external technology environment is a critical element for triggering students’ learning motivation (Chen, 2020). These results also suggested that perceived support from technology use is particularly relevant for students’ learning motivation and their attitude toward technology-based self-directed learning probably because they are confronted with the increasing technological modernity of the educational landscape (Lai et al., 2016).

Next, this study evaluated whether learning motivation is related to attitude toward technology-based self-directed learning. The results confirmed the links between learning motivation and attitude toward technology-based self-directed learning. Specifically, correlational coefficient (γ = 0.703, *p* < 0.01) and path analyses (β = 0.54, *p* < 0.01) showed that students with higher learning motivation also reported higher levels of attitude toward technology-based self-directed learning, indicating that learning motivation could be considered as an important antecedent for attitude toward technology-based self-directed learning (Elliot, 2006; Schunk and Pajares, 2009; Elliot and Hulleman, 2017). Importantly, our test of the relationships of constructs relies on the longitudinal data collected for a period of time after participants were instructed into technology use for language learning beyond class, which complements the literature of previous cross-sectional studies conducted in non-Eastern Asian samples.

Additionally, the study revealed that students’ learning motivation explained the associations between students’ perceptions of technology environments and their attitude toward technology-based self-directed learning. Specifically, learning motivation mediated the relations of technology acceptance, technological self-efficacy, and students’ attitude toward technology-based self-directed learning. Concretely, students perceiving greater technology use later reported higher levels of learning motivation, and in turn, students with higher levels of learning motivation also reported greater attitude toward technology-based self-directed learning. This finding added to evidence to the research from Fırat et al. (2018, p. 63), which emphasized motivation as one “of the most important factors affecting the speed, intensity, direction, and persistence of human behavior.” Overall, these findings confirmed that supportive technology environments exert positive influence on learning motivation, which in turn is an antecedent of learning attitude (Schunk and Pajares, 2009; Elliot and Hulleman, 2017). More importantly, this study provided the evidence concerning the explanatory mechanism of learning motivation for the relation between students’ simultaneous perceptions of technology acceptance and technological self-efficacy and their attitude toward technology-based self-directed learning.

This study adopted a longitudinal approach to explore the relation between technology environments and attitude toward technology-based self-directed learning and identifying specific paths from the technology acceptance and technological self-efficacy to attitude toward technology-based self-directed learning. The results of this study must be interpreted with some caution as several limitations exist. First, this study measured students’ perceived technology environments, learning attitude, and learning motivation through self-reported data, which may have affected the accuracy of the results. Future studies could combine other research methods (e.g., online learning observation, interviews) to verify the study results. Second, the latent influence of the behavior conducted by teachers and peers was neglected in this study. Future studies should empirically test models including other teachers’ or peers’ behaviors, such as teachers’ online feedback and peers’ interactivity and mutual evaluations, to assess their relevance to students’ attitude toward technology-based self-directed learning. Third, this study investigated the explanatory mechanism of learning motivation; however, other motivational factors, such as learning confidence, interest, or effort, might be taken into account for the relations between the technology acceptance and technological self-efficacy and attitude toward technology-based self-directed learning so as to deepen the understanding of the relations between the characteristics of technology environments and students’ attitude toward technology-based self-directed learning. Finally, learning motivation in this study was investigated as a general concept that influenced students’ adoption of technology for self-directed learning. It is suggested that the future research in this area would focus more detail on individual concept of learning motivation, analyze its dimensions more thoroughly (not as a single concept, but as a composite of many underlying concepts), and study them in a more concrete context (e.g., a specific type of learning, technology, etc.).

## Conclusion

At present, the development and application of technology have brought about innovations in the learning style of undergraduate students. In this educational landscape, this study explored the contribution of technology acceptance and technological self-efficacy to attitude toward technology-based self-directed learning in a sample of Chinese undergraduate students and also investigated whether learning motivation mediated these associations. The analysis of fitting results shows that in the process of technology environments contributing to attitude toward self-directed learning behavior, learning motivation significantly mediated this relationship. This result not only confirmed the theoretical hypothesis that technology environments contribute to the attitude toward self-directed learning behavior through learning motivation, but also revealed the internal mechanism of motivation contributing to the attitude of self-directed learning. On the one hand, learning motivation can promote learners’ attitude of self-directed learning. On the other hand, learning motivation can have a significant impact on the achievement of self-directed learning. Rotgans and Schmidt (2012) argued that learning motivation is indispensable as it directly affects learners’ perception of learning effectiveness and even strengthens learning behavior, thus playing a self-reinforcing role in the process of self-directed learning. The conclusions suggest optimizing the curriculum design, improving the role of technology in students’ learning, especially making more effective use of technology for self-directed language learning beyond class, and meanwhile stimulating students’ learning motivation.

## Data Availability Statement

The raw data supporting the conclusions of this article are available on request to the corresponding author.

## Ethics Statement

Ethical review and approval was not required for the study on human participants in accordance with the local legislation and institutional requirements. Written informed consent from the participants’ legal guardian/next of kin was not required to participate in this study in accordance with the national legislation and the institutional requirements.

## Author’s Note

XP is an associate professor in Xingzhi College, Zhejiang Normal University, China. His research interests are English Language teachers’ technology use for professional development and students’ learning, intercultural English education and educational psychology. His publications have appeared in *International Journal of Computer-assisted Language Learning and Teaching* and *Social Behavior and Personality*.

## Author Contributions

The author confirms being the sole contributor of this work and has approved it for publication.

### Conflict of Interest

The author declares that the research was conducted in the absence of any commercial or financial relationships that could be construed as a potential conflict of interest.
